# Supplementary material for: Evidence-Based Recommendations for an Optimal Prenatal Supplement for Women in the U.S., Part Two: Minerals
Source: Nutrients. 2021 May 28;13(6):1849. doi: 10.3390/nu13061849 (PMC8229801; doi:10.3390/nu13061849)
Supplement: Supplementary file 1 [file nutrients-13-01849-s001.zip › nutrients-1212354-supplementary.pdf]

Supplemental Table S1: Additional information about research studies discussed

Mineral(s) Reviewed	Reference	Country	Number of Participants	Study Type	Link
Calcium	Lerch 2007	Turkey, China, France	1700	O	https://pubmed.ncbi.nlm.nih.gov/17943890/
Calcium	Pettifor 2008			R	https://pubmed.ncbi.nlm.nih.gov/29450091/
Calcium	Wallis 2008	US	871856	O	https://pubmed.ncbi.nlm.nih.gov/18437143/
Calcium	Pitkin 1997	US	184	O	https://pubmed.ncbi.nlm.nih.gov/848531/
Calcium	Almaghamsi 2018			R	https://pubmed.ncbi.nlm.nih.gov/30410686/
Calcium	Kolthoff 1998	Croatia	59	T	https://pubmed.ncbi.nlm.nih.gov/9640346/
Calcium	He 2016		14 studies	M	https://pubmed.ncbi.nlm.nih.gov/26930501/
Calcium	Hofmeyr 2014	Worldwide	18064	T	https://pubmed.ncbi.nlm.nih.gov/30277579/
Calcium	Herrera 2005	Bangladesh, Colombia	48	T	https://pubmed.ncbi.nlm.nih.gov/16243339/
Calcium	Herrera 1998	Colombia	86	T	https://pubmed.ncbi.nlm.nih.gov/9540946/
Calcium	Mann 2010	US	87677	O	https://pubmed.ncbi.nlm.nih.gov/19936906/
Calcium	Li 2018	China	728	O/T	https://pubmed.ncbi.nlm.nih.gov/30593205/
Calcium	Bassaw 1998	Trinidad	510	T	https://pubmed.ncbi.nlm.nih.gov/15512028/
Calcium	Rogers 1999	China	500	T	https://pubmed.ncbi.nlm.nih.gov/10476618/
Calcium	Cong 1995	China	212	T	https://pubmed.ncbi.nlm.nih.gov/7712843/
Calcium	World 2011			R	https://pubmed.ncbi.nlm.nih.gov/23741776/
Chromium	Aharoni 1992	US	110	O	https://pubmed.ncbi.nlm.nih.gov/1728809/
Chromium	Hambidge 1969	US	21	O	https://pubmed.ncbi.nlm.nih.gov/5763119/
Chromium	Mahalko 1976	US	78	O	https://pubmed.ncbi.nlm.nih.gov/973597/
Chromium	Afridi 2009	Pakistan	144	O	https://pubmed.ncbi.nlm.nih.gov/19169697/
Chromium	Sundararaman 2012	India	30	O	https://pubmed.ncbi.nlm.nih.gov/22701850/
Chromium	Woods 2008	US	425	O	https://pubmed.ncbi.nlm.nih.gov/18343864/
Chromium	Jovanovic-Peterson 1996	US	24	T	https://pubmed.ncbi.nlm.nih.gov/8632110/
Chromium	Jamilian 2016	Iran	60	T	https://pubmed.ncbi.nlm.nih.gov/26613790/
Chromium	Amiri Siavashani 2018	Iran	40	T	https://pubmed.ncbi.nlm.nih.gov/30546347/
Chromium	Suksomboon 2014		1498	M	https://pubmed.ncbi.nlm.nih.gov/24635480/
Chromium	Iovieno 2011	US		R	https://pubmed.ncbi.nlm.nih.gov/20579741/
Copper	Alebic-Juretic 2005	Croatia	319	O	https://pubmed.ncbi.nlm.nih.gov/16325535/
Copper	Vukelic 2012	Serbia	2170	O	https://pubmed.ncbi.nlm.nih.gov/22462346/
Copper	Buamah 1984		259	O	https://pubmed.ncbi.nlm.nih.gov/6478599/
Copper	Morton 1976	South Wales	20	O	https://pubmed.ncbi.nlm.nih.gov/949571/
Copper	Zhang 2006	Europe	100	O	https://europepmc.org/article/med/16635319
Copper	Song 2017		1360	M	https://pubmed.ncbi.nlm.nih.gov/28385285/
Copper	Fan 2016		905	M	https://pubmed.ncbi.nlm.nih.gov/27407173/
Copper	Thauvin 1992	France	62	T	https://pubmed.ncbi.nlm.nih.gov/1375082/
Iodine	Borekci 2009	Turkey	40	O	https://pubmed.ncbi.nlm.nih.gov/19034391/
Iodine	Adams 2006	US	145	O	https://pubmed.ncbi.nlm.nih.gov/16845157/
Iodine	Adams 2011	US	99	O	https://pubmed.ncbi.nlm.nih.gov/21651783/
Iodine	de Escobar 2007			R	https://pubmed.ncbi.nlm.nih.gov/18053280/
Iodine	de Benoist			R	https://pubmed.ncbi.nlm.nih.gov/18947032/
Iodine	Leung 2012			R	https://pubmed.ncbi.nlm.nih.gov/?term=Leung%2C+2012%2C+History+of+U.S.+iodine+fortification+and+supplementation&filter=

					simsearch3.fft
Iodine	Hollowell 1998	US	62818	O	https://pubmed.ncbi.nlm.nih.gov/9768638/
Iodine	Caldwell 2013	US	501	O	https://pubmed.ncbi.nlm.nih.gov/23488982/
Iodine	Glinoer 1997			R	https://pubmed.ncbi.nlm.nih.gov/9183570/
Iodine	Lieberman 1998	Chile	16	O	https://pubmed.ncbi.nlm.nih.gov/9768662/
Iodine	Brander 2003	Switzerland	316	O	https://pubmed.ncbi.nlm.nih.gov/12906364/
Iodine	Alexander 2017			R	https://pubmed.ncbi.nlm.nih.gov/28056690/
Iodine	Delange 2007			R	https://pubmed.ncbi.nlm.nih.gov/18053281/
Iodine	Berbel 2009	Spain	345	O	https://pubmed.ncbi.nlm.nih.gov/19348584/
Iodine	O'Donnell 2002	China	207	O	https://pubmed.ncbi.nlm.nih.gov/11848114/
Iodine	Delange 2005			R	https://pubmed.ncbi.nlm.nih.gov/16398455/
Iodine	Moreno-Reyes 2013	Belgium	1311	O	https://pubmed.ncbi.nlm.nih.gov/23846819/
Iodine	Vermiglio 2004	Italy	27	O	https://pubmed.ncbi.nlm.nih.gov/15579758/
Iodine	Bath 2013	UK	1040	O	https://pubmed.ncbi.nlm.nih.gov/23706508/
Iodine	Hynes 2013	Australia	228	O	https://pubmed.ncbi.nlm.nih.gov/23633204/
Iodine	van Mil 2012	Netherlands	1156	O	https://pubmed.ncbi.nlm.nih.gov/23077186/
Iodine	Levie 2018	Spain, Netherlands, UK	9036	M	https://pubmed.ncbi.nlm.nih.gov/29757392/
Iodine	Dineva 2020		37 studies	R/M	https://pubmed.ncbi.nlm.nih.gov/32320029/
Iodine	Drover 2019		28 studies	R	https://pubmed.ncbi.nlm.nih.gov/30299402/
Iodine	Borekci 2009	Turkey	40	O	https://pubmed.ncbi.nlm.nih.gov/19034391/
Iodine	Censi 2019	Italy	90	T	https://pubmed.ncbi.nlm.nih.gov/31689890/
Iodine	Antonangeli 2002	Italy	67	T	https://pubmed.ncbi.nlm.nih.gov/12088916/
Iodine	Markhus 2018	Norway	851	O	https://pubmed.ncbi.nlm.nih.gov/30205599/
Iron	Cogswell 2003	US	513	T	https://pubmed.ncbi.nlm.nih.gov/14522736/
Iron	Siega-Riz 2006	US	867	T	https://pubmed.ncbi.nlm.nih.gov/16458655/
Iron	Gupta 2017	US	5316	O	https://pubmed.ncbi.nlm.nih.gov/29070559/
Iron	Schmidt 2014	US	866	O	https://pubmed.ncbi.nlm.nih.gov/25249546/
Iron	Krapels 2004	Netherlands	355	O	https://pubmed.ncbi.nlm.nih.gov/15514283/
Iron	Chandler 2012	US	7222	O	https://pubmed.ncbi.nlm.nih.gov/22933447/
Iron	Makrides 2003	Australia	430	T	https://pubmed.ncbi.nlm.nih.gov/12816784/
Iron	Meier 2003	US	111	T	https://pubmed.ncbi.nlm.nih.gov/15931282/
Iron	Preziosi 1997	Niger	197	T	https://pubmed.ncbi.nlm.nih.gov/9356536/
Iron	Kilbride 1999	Jordan	232	O	https://pubmed.ncbi.nlm.nih.gov/10405849/
Iron	Scholl 2011			R	https://pubmed.ncbi.nlm.nih.gov/22043878/
Iron	Pena-Rosas 2009		23200	R	https://pubmed.ncbi.nlm.nih.gov/19821332/
Iron	Pena-Rosas 2015		43274	M	https://pubmed.ncbi.nlm.nih.gov/26198451/
Magnesium	De Jorge 1965	Brazil	139	O	https://pubmed.ncbi.nlm.nih.gov/14268597/
Magnesium	Standley 1997	US	31	O	https://pubmed.ncbi.nlm.nih.gov/8990431/
Magnesium	Hall 1957	US	324	O	https://pubmed.ncbi.nlm.nih.gov/13400427/
Magnesium	Arikan 1999	Austria	209	O	https://pubmed.ncbi.nlm.nih.gov/10640870/
Magnesium	Zarcone 1994	Italy		T	https://pubmed.ncbi.nlm.nih.gov/7603732/
Magnesium	Jafrin 2014	Bangladesh	108	O	https://pubmed.ncbi.nlm.nih.gov/25481580/
Magnesium	Tayana 2013	Iran	500	O	https://pubmed.ncbi.nlm.nih.gov/24693379/
Magnesium	Enaruna 2013	Nigeria	160	O	https://pubmed.ncbi.nlm.nih.gov/23974737/
Magnesium	Uludag 2014	Turkey	100	T	https://pubmed.ncbi.nlm.nih.gov/24156667/
Magnesium	He 2016		14 studies	M	https://pubmed.ncbi.nlm.nih.gov/26930501/
Magnesium	Krapels 2004	Netherlands	355	O	https://pubmed.ncbi.nlm.nih.gov/15514283/
Magnesium	Markrides 2014		9090	M/T	https://pubmed.ncbi.nlm.nih.gov/24696187/
Magnesium	Harrison 2007	South Africa	4494	T	https://pubmed.ncbi.nlm.nih.gov/17578470/

Magnesium	D'Almeida 1992	Angola	150	T	https://pubmed.ncbi.nlm.nih.gov/1492408/
Magnesium	Li 1997	China	102	T	https://pubmed.ncbi.nlm.nih.gov/9642379/
Magnesium	Arikan 1997	Austria	481	T	https://www-sciencedirect-com.ezproxy1.lib.asu.edu/science/article/pii/S0002937897801925
Magnesium	Zarcone 1994	Italy	100	T	https://pubmed.ncbi.nlm.nih.gov/7603732/
Magnesium	Spatling 1998	Switzerland	568	T	https://pubmed.ncbi.nlm.nih.gov/3349001/
Magnesium	Kovacs 1998	Germany	985	T	https://pubmed.ncbi.nlm.nih.gov/3063587/
Magnesium	Sibai 1989	US	374	T	https://pubmed.ncbi.nlm.nih.gov/2665492/
Magnesium	Doyle 2009		6145	M	https://pubmed.ncbi.nlm.nih.gov/19160238/
Manganese	Spencer 1999	Australia	34	O	https://pubmed.ncbi.nlm.nih.gov/10501283/
Manganese	Vigeh 2008	Iran	410	O	https://pubmed.ncbi.nlm.nih.gov/18242051/
Manganese	Zota 2009	US	470	O	https://pubmed.ncbi.nlm.nih.gov/19289966/
Manganese	Than 2009	US	124	O	https://pubmed.ncbi.nlm.nih.gov/19900038/
Manganese	Eum 2014	Korea	331	O	https://pubmed.ncbi.nlm.nih.gov/24775401/
Manganese	Mora 2015	Costa Rica	380	O	https://pubmed.ncbi.nlm.nih.gov/25460620/
Manganese	Jones 2010	US	102	O	https://pubmed.ncbi.nlm.nih.gov/20553999/
Manganese	Chung 2015	Korea	232	O	https://pubmed.ncbi.nlm.nih.gov/25734517/
Manganese	Lin 2013	Taiwan	230	O	https://pubmed.ncbi.nlm.nih.gov/23578827/
Molybdenum	Waring 2000	UK	300	O	https://www.tandfonline.com/doi/abs/10.1080/13590840050000861
Molybdenum	Vazquez-Salas 2014	Mexico	147	O	https://pubmed.ncbi.nlm.nih.gov/24479423/
Selenium	Zachara 1993	Poland	49	O	https://pubmed.ncbi.nlm.nih.gov/8449427/
Selenium	Rayman 2014	UK	230	T	https://pubmed.ncbi.nlm.nih.gov/24708917/
Selenium	Choi 2016	Korea	245	O	https://pubmed.ncbi.nlm.nih.gov/27886083/
Selenium	Varsi 2017	Norway	272	O	https://pubmed.ncbi.nlm.nih.gov/28492511/
Selenium	Kong 2016	Global	569	M	https://pubmed.ncbi.nlm.nih.gov/27793207/
Selenium	Mariath 2011	Brazil		R	https://pubmed.ncbi.nlm.nih.gov/21338537/
Selenium	Bro 1988	Denmark	500	O	https://pubmed.ncbi.nlm.nih.gov/2980811/
Selenium	Rayman 2014	UK	230	O	https://pubmed.ncbi.nlm.nih.gov/24708917/
Selenium	Mokhber 2011	Iran	169	T	https://pubmed.ncbi.nlm.nih.gov/20528216/
Selenium	Negro 2007	Italy	2143	T	https://pubmed.ncbi.nlm.nih.gov/17284630/
Zinc	Hambidge 2017	Guatemala	600	O	https://pubmed.ncbi.nlm.nih.gov/28424260/
Zinc	Hunt 1983	US	213	T	https://pubmed.ncbi.nlm.nih.gov/6837492/
Zinc	Goldenberg 1995	US	580	T	https://pubmed.ncbi.nlm.nih.gov/7629954/
Zinc	Hambidge 1983	US	46	T	https://pubmed.ncbi.nlm.nih.gov/6829485/
Zinc	Tamura 2000	US	3448	O	https://pubmed.ncbi.nlm.nih.gov/10617954/
Zinc	Xie 2001	China	156	T	
Zinc	Huang 1999	China	136	O	https://pubmed.ncbi.nlm.nih.gov/10433344/
Zinc	Ilhan 2002	Turkey	75	O	https://pubmed.ncbi.nlm.nih.gov/12270770/
Zinc	Choi 2016	Korea	245	O	https://pubmed.ncbi.nlm.nih.gov/27886083/
Zinc	Ma 2015	Global	1425	M/O	https://pubmed.ncbi.nlm.nih.gov/26389947/
Zinc	He 2016	Global	14 studies	M	https://pubmed.ncbi.nlm.nih.gov/26930501/
Zinc	Beckhaus 2015	Global	32 studies	M	https://pubmed.ncbi.nlm.nih.gov/26296633/
Zinc	Devereux 2006	UK	1861	O	https://pubmed.ncbi.nlm.nih.gov/16763215/
Zinc	Mukherjee 1984		450	O	https://pubmed.ncbi.nlm.nih.gov/6475821/
Zinc	Lazebnik 1988	US	279	O	https://pubmed.ncbi.nlm.nih.gov/3337165/
Zinc	Hunt 1985	US	138	T	https://pubmed.ncbi.nlm.nih.gov/4061343/
Zinc	Ota 2015	Global	17000	M	https://pubmed.ncbi.nlm.nih.gov/25927101/
Zinc	Tamura 2003	US	355	T	https://pubmed.ncbi.nlm.nih.gov/12791632/
Zinc	Darmstadt 2012	Bangladesh	559	T	https://pubmed.ncbi.nlm.nih.gov/22189527/
Zinc	Bowen 2015	Global	145,028	R	https://pubmed.ncbi.nlm.nih.gov/26317533/
Zinc	Xie 2001	China	156	T	

Supplemental Table S1 gives additional information about the studies included. M represents a meta-analysis, R represents reviews, O represents observational study and T represents a treatment study.
